# Developmental Impact of Maternal Immune Activation on the Fetal Immune System and Lung

**DOI:** 10.1002/eji.70138

**Published:** 2026-01-26

**Authors:** Walaa Jradi, Kira Duhm, Clarissa Prazeres da Costa

**Affiliations:** ^1^ Department of Preclinical Medicine, Institute for Medical Microbiology, Immunology and Hygiene (MIH), TUM School of Medicine and Health Technical University of Munich (TUM) Munich Germany; ^2^ Center for Global Health, TUM School of Medicine and Health Technical University of Munich Munich Germany; ^3^ German Center for Infection Research (DZIF) Partner Site Munich Munich Germany

**Keywords:** allergy, hematopoiesis, immune system, lung development, maternal immune activation (MIA), microbiota

## Abstract

Maternal immune activation (MIA) refers to an immune response triggered in a pregnant mother by infections, inflammation, or other immune challenges that can impact offspring health. We propose aligning MIA within the framework of the developmental origins of health and disease (DOHaD) theory because it has the potential to provide mechanistic evidence for long‐term outcomes of fetomaternal crosstalk disruptions. MIA models are created by exposing pregnant animals to immune‐activating agents such as inosinic–polycytidylic acid (poly I:C) or lipopolysaccharide (LPS), which mimic viral or bacterial infections, respectively. Next to these acute MIA models, chronic helminth infections during pregnancy have been employed as an additional, more physiological model of infection. MIA models have helped researchers explore how maternal infections during pregnancy may impact the offspring's risk of neurodevelopmental disorders. Emerging evidence suggests that these models have a broader impact on organ development, the immune system, and, consequently, immune‐related disorders such as allergies. Our review focuses on evidence derived mainly from mouse models of MIA that have investigated maternal signals, such as cytokines and microbiota, on fetal hematopoiesis, adults’ immune cell compartments, including the bone marrow, and their relation to the development of offspring allergies. Where applicable, studies from other species are indicated.

## Graphical abstract

Maternal immune activation triggers elevated cytokines (IL‐6 and IL‐17A), disrupting fetal immune system development. This not only alters hematopoietic stem cell balance but also leads to ILC2 hyperactivation in the lung, which could potentially affect susceptibility to allergic diseases in the offspring.

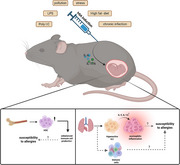

**Abbreviations**
AGM: aorta–gonad–mesonephros
ASD: autism spectrum disorder
CORT: corticosterone
DOHad: developmental origins of health and disease
E: embryonic day
EMPs: erythro‐myeloid progenitors
GM‐CSF: granulocyte–macrophage colony‐stimulating factor
HSCs: hematopoietic stem cells
IFN‐γ: interferon gamma
Ig: immunoglobulin
IL: interleukin
ILCs: innate lymphoid cells
LPS: lipopolysaccharide
MIA: maternal immune activation
OVA: ovalbumin
PolyI:C: polyinosinic:polycytidylic acid
Th2: T helper type 2 cells
TLR: toll‐like receptor

## 1 The Concept of Maternal Immune Activation (MIA) and Its Relatedness to the Developmental Origins of Health and Disease (DOHaD) Theory Framework

The DOHaD theory provides a biosocial framework exploring how environmental factors during early development—such as prenatal and perinatal nutrition, stress, chemical exposure, or infections—impact long‐term health outcomes [1, 2, 3]. Originating from findings in human epidemiological studies that showed strong associations between birthweight and rates of adult death from ischemic heart diseases by David Barker et al. [4], further support came from the Dutch Hunger Winter studies that consequently demonstrated that maternal undernutrition enhanced the risk of non‐communicable diseases like cardiovascular diseases as well as diabetes during adulthood [5, 6]. Indeed, only recently, a human cohort study found that sugar rationing in the first 1000 days of life has been found to reduce the risk of type 2 diabetes and hypertension by 35% and 20%, respectively [7]. Overall, the critical window is characterized by the rapid development of organs and tissues but also by the first social experiences within the first 1000 days of life, from conception to 2 years after birth. Exploration of underlying mechanisms for such long‐term impacts during this period is obviously challenging and has largely focused on investigations of epigenetic gene regulation in past years, with some potential explanations summarized here [8]. However, large retrospective methylation studies of various tissues, cell types, and genes have complicated the power and comparability of the findings [9]. They have nonetheless supported the generation of the emerging field of population epigenetics to generate robust and reliable evidence to explore causality and functional consequences of what is sometimes also called “fetal programming.” Another concept that is gaining momentum in the field of the science of early life events is that of MIA. This term was coined in 2005 in order to describe the observations that inflammatory processes during pregnancy, such as influenza infection, could lead to neurodevelopmental disorders such as autism spectrum disorder (ASD) in children and young adults, schizophrenia, and potentially dementia later in life [5, 10, 11]. Interestingly, immune‐system‐related disorders, such as elevated levels of monocytes and plasma cytokines, are more frequently observed in individuals with neuropsychiatric disorders compared to matched healthy controls [12, 13]. Whether these are causal for the disease development or its severity or constitute secondary consequences of the disease itself remains unclear. This naturally calls for disease models, such as those in animals, to study the causes and consequences, including downstream mechanisms leading to disease. Furthermore, by linking MIA with DOHaD, researchers are gaining insights into how maternal health during pregnancy contributes to immune‐system‐related outcomes in offspring, such as allergies and asthma, emphasizing the importance of maternal immune status [14]. Thus, in this review, we summarize evidence derived from studies performed mainly in mice to explore how events in utero, including alterations in the maternal microbiota, are related to the development of the immune system, mainly in regard to hematopoiesis as well as the lung (Table 1).

**TABLE 1:** Summary of selected studies investigating the effects of maternal immune activation (MIA) on offspring development.

Reference	MIA trigger	Time point	Cells/Organs affected	Outcome in offspring
Choi et al. [20]	Poly(I:C), IL‐17A	E12.5	Brain (cortical development)	Autism‐like behaviors; cortical abnormalities
Dehmel et al. [26]	Intrauterine smoke	Throughout pregnancy	Lung	Dysregulation in the pulmonary transcriptome, postnatal growth retardation, and reduction in lung function
Edlow et al. [29]	HFD	12–14 weeks prior to mating	Fetal brain	Disrupted fetal brain gene expression in sex sex‐specific manner
Shen et al. [33]	Poly(I:C)	E12.5	Maternal T_regs_ and macrophages	Autism‐like behaviors reversed by macrophage depletion
Church et al. [36]	Repeated OVA exposure at early gestation	GD2‐9	Brain	Male‐specific behavioral deficits
Lim et al. [44]	*Yersinia pseudotuberculosis*, IL‐6	E10.5	Intestine (Th17 cells, IECs)	Increased Th17 cells; enhanced colitis susceptibility
Kim et al. [47]	Poly(I:C), SFB microbiota	E12.5	Brain	ASD‐like symptoms; SFB involvement
Kim et al. [48]	Poly(I:C), altered microbiota	E12.5	CD4+ T cells (chromatin)	Increased intestinal inflammation, IL‐17A+ T cells
Lima et al. [41]	IFN‐γ treatment during gestation	GD6.5	Lung (eosinophils, goblet cells)	Reduced allergic sensitization and Th2 cytokines
Straubinger et al. [42]	Chronic schistosoma infection	Timely infected before mating	Airway (eosinophils)	Allergy protection dependent on maternal Th1 phase
Lacorcia et al. [43]	Chronic helminth infection	16 weeks before mating	DCs, T cells	Altered DC function, reduced T cell priming
Smith et al. [49]	Maternal CORT	E12.5	Lung (goblet cells, immune infiltrates)	Sex‐dependent allergic inflammation
Victor et al. [50]	Maternal OVA immunization	Immunized before mating	B cells (FcγRIIb), Th2 cells	Reduced IgE, Th2 cytokines, allergy modulation
Conrad et al. [51]	*Acinetobacter lwoffii* F78 (microbe)	Timely treatment before mating	Airway (eosinophils, goblet cells)	Asthma protection via maternal TLR signaling
Brand et al. [52]	*A. lwoffii* F78 (microbe)	Timely treatment before mating	CD4+ T cells (histone acetylation)	Epigenetic asthma protection; reversed by HAT inhibitor
Lopez et al. [58]	Poly(I:C)	E14.5	Fetal HSPCs, MPPs (bone marrow)	Expansion of lymphoid progenitors; altered immunity
Lopez et al. [81]	Poly(I:C)	E14.5	Lung ILC2s	ILC2 hyperactivation; increased asthma susceptibility
Lopez and Beaudin [80]	Poly(I:C)	E14.5	Lung ILC2s	Expansion and hyperactivation of ILC2s

*Note*: The table highlights the experimental model or immune trigger used to induce MIA at specific time points, the specific organs or immune cell types affected (e.g., bone marrow, lung, gut, and brain), and the corresponding outcomes observed in the offspring, including behavioral changes, immune cell programming, allergic susceptibility, and microbiota‐related effects. This summary reflects the primary focus areas discussed throughout the review.

Abbreviations: ASD, autism spectrum disorder; CORT, corticosterone; IFN‐γ, interferon‐gamma; OVA, ovalbumin; SFB, segmented filamentous bacteria; TLR, toll‐like receptor.

## 2 MIA: Triggers and Role of Maternal Cytokines

MIA can be experimentally induced by several factors, including infectious and noninfectious stimuli, as well as environmental factors. Infectious stimuli, such as viral infections, including influenza virus and severe acute respiratory syndrome coronavirus 2, have been shown to induce behavioral deficits resembling ASD and schizophrenia in offspring of both rodent and nonhuman primate models [15, 16, 17]. Pathogen‐associated molecular patterns, such as poly(I:C), lipopolysaccharide (LPS), and cytosine–phosphate–guanine, all of which are toll‐like receptor (TLR) agonists, are commonly used to trigger MIA in experimental settings in order to recreate the effects of actual infections. Over the years, these models have provided valuable insights into the link between MIA and neurodevelopmental disorders. For instance, injection of poly(I:C) or LPS during pregnancy has been shown to induce ASD in offspring. Sex‐specific molecular changes in inflammation‐related genes, such as in the brain, have also been documented. These results are in line with the observation that ASD‐like symptoms are more common in male children born to mothers experiencing influenza infection during pregnancy [18, 19, 20, 21, 22, 23].

In addition, noninfectious factors, such as smoking or other environmental pollutants like diesel particles, nutrition, or stress, are well‐known inducers of maternal immune responses. These factors increase pro‐inflammatory cytokines such as tumor necrosis factor alpha, interleukin‐6 (IL‐6), and IL‐1 beta [24]. In particular, human studies have reported that cigarette smoke exposure elevates these cytokines, although results have been inconsistent across different cohorts [25]. In addition, maternal smoking plays an important role in impacting fetal lung development and long‐term respiratory health. Studies have shown that intrauterine smoke exposure leads to dysregulation of the pulmonary transcriptome, postnatal growth retardation, and reduction in lung function in a sex‐specific manner in young offspring, with insulin‐like growth factor 1 emerging as a key regulatory factor. These findings suggest that prenatal smoke exposure may not only impair lung development but also predispose offspring to chronic lung diseases, possibly through persistent epigenetic modifications and immune dysregulation [26, 27]. Other environmental factors, such as exposure to airborne particulate matter, especially with a particulate matter size of 2.5 µm, during the third trimester, have been associated with an increased risk of ASD development in early childhood [28].

Additionally, maternal obesity and high‐fat diets during pregnancy have been shown to disrupt fetal brain gene expression in a sex‐dependent manner [29] and induce placental immune inflammation [30] with an increase in pro‐inflammatory cytokines such as IL‐6 and IL‐1 beta [31]. Studies have also shown a shift in placental macrophages towards a pro‐inflammatory phenotype, leading to impaired trophoblast function and disrupted nutrient exchange. Consequently, these disruptions contributed to behavioral changes in offspring, such as anxiety, impaired sociability, and cognitive deficits [31, 32]. Interestingly, later investigations confirmed that a poly(I:C) model of MIA also induced proinflammatory F4/80^+^CD86^+^ rather than anti‐inflammatory F4/80^+^CD206^+^ macrophages and reduced T regulatory cells in the mothers, which were collectively responsible for the behavioral abnormalities later in the adult offspring; maternal macrophage depletion, on the other hand, reversed these behavioral abnormalities in these offspring [33]. Consistent with the evolving understanding of macrophage biology, recent studies focus on the limitations of the binary M1/M2 classification and instead refer to a spectrum of activation states defined by transcriptional and epigenetic profiles in mouse and human macrophages [34]. This paradigm shift is indicative of the plasticity of macrophages, similar to T cells, and reflects their capacity to dynamically adapt to tissue‐specific signals and inflammatory stimuli. Furthermore, maternal stress in pregnant women, including experiences of trauma (such as emotional, physical, and sexual abuse and emotional and physical neglect), especially during the early stages of pregnancy, has an increasing influence on neurodevelopmental outcomes but is dependent on the immunological response [35]. Further studies have also shown that allergic airway inflammation induced by repeated exposures to ovalbumin (OVA) during pregnancy leads to behavioral abnormalities in adult offspring, such as increased anxiety and repetitive behaviors. These effects were sex‐dependent, with male offspring showing more pronounced behavioral changes [36, 37]. These diverse stimuli often do not occur in isolation but can interact to generate a complex and multifaceted impact on MIA and subsequent offspring organ development [38].

Studies investigating the underlying mechanisms of such MIA‐induced effects have focused on maternal cytokines as mediators that could potentially pass the placenta or elicit lasting effects within the placenta. Elevated maternal IL‐6 levels can disrupt the placental microenvironment, altering nutrient and oxygen exchange and impairing fetal growth [39, 40]. Other studies have shown that applying interferon‐gamma (IFN‐γ) during gestation suppressed allergic inflammation in offspring by reducing eosinophilic infiltration and goblet cell hyperplasia. Prenatal IFN‐γ exposure also shifted immune programming away from a T helper (Th)2‐dominant response, leading to lower IL‐4 and IL‐5 production, reduced plasma immunoglobulin (Ig)E and IgG1, and decreased allergic sensitization. This effect was most potent when exposure to IFN‐γ occurred in utero rather than postnatally when offspring were cross‐fostered, highlighting the importance of early fetal immune conditioning. A proposed hypothesis was that IFN‐γ may mediate these changes by modulating maternal antigen‐presenting cells, such as dendritic cells and macrophages, ultimately biasing offspring immunity towards an anti‐allergic state [41]. Interestingly, using a model of chronic schistosomiasis, it was shown that maternal infection during pregnancy can protect offspring from allergic airway inflammation in an immune phase‐dependent manner. Protection in adult offspring was observed during the maternal IFN‐γ‐prone Th1 phase of infection but was lost in mothers deficient in IFN‐γ secretion [42]. These findings support the role of maternal immune modulation, resulting in certain cytokine profiles that shape offspring allergy susceptibility. The question remains, though, which parts of the offspring immune system—adaptive and/or innate—are altered by these exposures or whether these are already imprinted at the stem cell level. Indeed, studies using the chronic helminth infection model have shown that maternal infections can induce diverse and long‐lasting changes in adult offspring immune system. These changes included enhanced major histocompatibility complex class II and CD86 expression in conventional dendritic cells and mature B cells, along with the reduced capacity of these dendritic cells to induce antigen‐specific T cell proliferation [43].

It is important to note that MIA does not yield universally deleterious or beneficial outcomes. For example, as discussed earlier, although influenza infection during pregnancy has been associated with neurodevelopmental disorders such as ASD in offspring, IFN‐γ exposure has been shown to suppress allergic inflammation. These seemingly contradictory effects highlight the complexity and context dependency of maternal immune exposures, which depend on timing, duration, and the immune pathway involved.

Furthermore, as mentioned previously, cytokines that cross the placenta could directly influence progenitor cells and fetal tissue development. Only recently, a study showed that maternal IL‐6 is sensed by offspring basal crypt stem cells in the intestine, which continuously give rise to functional intestinal epithelial cells. Lack of the IL‐6 receptor in intestinal cells significantly reduced Th17 responses in the offspring gut [44]. Such systemic changes may thus skew immune system programming and hematopoietic trajectories, ultimately affecting the function and composition of immune cells at birth. Moreover, organs like the fetal lung, which play a dual role in hematopoietic development and immune system establishment, are particularly vulnerable to inflammatory perturbations and, thus, potentially also to MIA.

## 3 Mechanistic Insights Into How MIA Programs Offspring Immunity: From Cytokines to Microbiota

Increasing evidence demonstrates how the presence and composition of microbiota can significantly impact immune responses. One example is the study on commensal intestinal bacteria, which identified SFB (segmented filamentous bacteria) as a key inducer of Th17‐cell differentiation in the gut. Colonization with SFB led to the accumulation of CD4+ T cells with effector functions, particularly Th17 cells, in the gut lamina propria [45]. Thus, it is important to highlight the influence of maternal microbiota shifts during MIA on offspring immune system development. Maternal cytokines and alterations in maternal microbiota during MIA likely cooperate to drive changes in offspring behavior and immune development.

At steady state, the role of maternal microbiota in programming offspring immunity was investigated by showing that maternal colonization with a defined microbial community elevated ILC3s and F4/80(+)CD11c(+) mononuclear cells in offspring and enhanced the expression of genes involved in antibacterial peptide production and microbial metabolism in the intestine [46]. In the context of MIA, another study confirmed that SFB is not only crucial for inducing intestinal Th17 cells but is also responsible for MIA‐associated behavioral and brain abnormalities. This was confirmed by pretreating pregnant mothers with vancomycin before poly(I:C) injection at E12.5, which reduced SFB colonization in the mothers and abolished cortical abnormalities in the offspring brain, thus linking maternal immune status with offspring outcomes such as cortical abnormalities and behavioral deficits associated with ASD [47]. Later, studies showed that the offspring of pregnant mice exposed to elevated IL‐17A levels due to poly(I:C) administration at E12.5 exhibited increased susceptibility to intestinal inflammation later in life. This immune‐primed phenotype was mechanistically linked to changes in the maternal gut microbiota, with the alpha diversity (a measure of species richness and evenness) being diminished in MIA mothers and the beta diversity (a statistic used to quantify the compositional dissimilarity) substantially differing between MIA and phosphate‐buffered saline (PBS) mothers. They further demonstrated that stool transfer from IL‐17A‐primed pregnant mice into germ‐free dams recapitulated the immune‐primed phenotypes in offspring; specifically, the offspring exhibited enhanced inflammatory responses and higher levels of IL‐17A‐producing T cells. They also showed that mono‐colonization with SFB in poly(I:C)‐injected mothers was able to induce ASD in the offspring; however, the offspring didn't show changes in the percentages of IL‐17A and IFN‐γ‐producing T cells, confirming the SFB contribution to IL‐17A‐driven neurodevelopmental changes but not the immunological phenotypes in offspring. This suggests that rather than a single bacterial species, an altered gut microbial community is necessary for the emergence of long‐term immune‐primed phenotypes in MIA offspring. Finally, they linked the MIA‐induced changes in the maternal microbial community and IL‐17A to primed CD4+ T cells in offspring; they showed that altered maternal microbiota promoted immune priming by affecting T cells’ chromatin accessibility [48]. Similarly, prenatal maternal exposure to a bacterial infection with *Yersinia pseudotuberculosis* resulted in elevated intestinal Th17 cells in offspring, mediated by IL‐6 signaling in the fetal intestinal epithelium with maternal IL‐6 potentially crossing the placenta. Although this conferred enhanced resistance in offspring to enteric pathogens like *Salmonella typhimurium*, it also heightened susceptibility to inflammatory diseases, such as colitis, underscoring the dual‐edged nature of MIA‐induced immune modulation [44].

## 4 MIA: Shaping Offspring Susceptibility to Allergic Diseases

MIA alterations of the immune system's development can affect the susceptibility of offspring to immune‐driven diseases such as allergies. Allergic asthma, a chronic inflammatory disorder of the airways, can be modulated by different prenatal exposures during pregnancy. As mentioned previously, chronic maternal schistosomiasis caused long‐lasting immune modulation and protected offspring from allergic airway inflammation, but this depended on the infection phase [42, 43]. Additionally, studies have shown that chronic heightened maternal stress hormone corticosterone (CORT) led to sex‐dependent effects on allergic inflammation in adult offspring. Male offspring from CORT‐treated dams had an increased number of inflammatory cells in the lung in response to house dust mite treatment, whereas female immune cell numbers remained unaffected. However, maternal CORT following house dust mite caused heightened goblet cell hyperplasia in female but not male offspring. Thus, maternal stress had sexually dimorphic effects on allergic inflammation in the airways of adult offspring [49]. Another study on maternal immunization with OVA revealed that MIA before or during pregnancy could modulate the immune response in offspring. They showed that maternal OVA immunization increased Fcy‐RIIb, an inhibitory IgG receptor, expression in newborn and young offspring B cells, contributing to a reduction in Th2 responses and OVA‐specific IgE production, two key features of allergy. Antigen (Ag)‐specific IgG antibody was transferred to the offspring by transplacental and breastfeeding routes. The upregulation of FcγRIIb on splenic B cells then allowed for the cross‐linking of the receptor through IgG‐Ag complexes, eventually inhibiting B cell proliferation and activation. Furthermore, maternal immunization exerted a down‐modulatory effect on both IL‐4‐ and IFN‐γ‐secreting T cells and IL‐4‐ and IL‐12‐secreting B cells, contributing to an overall reduction in the Th2/Th1 cytokine levels [50].

However, the role of maternally transferred antigen‐specific antibodies may vary depending on immune conditions. Unpublished data using a neonatal RSV infection model indicate that maternal allergen‐specific antibodies, rather than suppressing allergy, can promote allergic Th2 responses in the offspring. This effect is mediated by the activation of Fc receptors on dendritic cells in the presence of viral infection. These findings contrast with earlier studies showing a protective role of maternal antibodies and highlight a need for further investigation into the factors that determine whether maternal antibodies confer protection or promote sensitization.

Further research supported the idea that exposure to microbial agents during pregnancy can play an essential role in shaping allergic responses in offspring postnatally. Conrad et al. showed that chronic maternal airway exposure to *Acinetobacter lwoffii* F78, a cowshed‐derived bacterium, prevented asthma development in adult OVA‐sensitized and challenged offspring; this was associated with a decrease in eosinophils and lymphocytes infiltration into the airways and fewer mucus‐producing goblet cells. Maternal TLR responses were crucial, as TLR2/3/4/7/9−/− mice showed no immune activation. Furthermore, maternal TLR dysfunction reversed the asthma‐protective effects of prenatal *A. lwoffii* exposure in offspring, highlighting the essential role of maternal TLR signaling in the placenta to mediate allergy protection in offspring [51]. More research showed that blocking IFN‐γ function, using a neutralizing monoclonal antibody (anti‐IFN‐γ mAb), in the offspring of *A. lwoffii* F78‐treated mothers restored the inflammation in the airway. Additionally, histone modification in offspring CD4+ T cells, specifically H4 acetylation at the IFNG locus, was linked to increased IFN‐γ levels in offspring as a potential protective mechanism. Treatment with garcinol, an inhibitor of histone acetyltransferases, prevented acetylation changes at the IFNG promoter and reversed asthma protection. These findings suggest that histone acetylation at the IFNG locus may be crucial for asthma protection by microbial exposures, though further studies are needed to investigate other epigenetic modifications involved [52].

## 5 MIA and Critical Windows for the Development of the Immune System

### 5.1 Hematopoietic Stem Cell (HSC) Development in Yolk Sac, Aorta–Gonad–Mesonephros (AGM), and Fetal Liver

As already discussed, MIA has long‐lasting consequences on offspring immunity, affecting their susceptibility to immune‐driven diseases. These persistent effects are observed well into adulthood, raising the question if prenatal inflammatory exposures might disrupt the earliest stages of immune system development. MIA may alter the differentiation and programming of hematopoietic stem and progenitor cells rather than influencing only mature immune cells. To better understand how MIA could induce such long‐term immune changes, it is essential to review the timeline and microenvironments of hematopoietic development during fetal life, particularly in the fetal liver, bone marrow, and potentially the lung, and how these niches might be vulnerable to inflammatory cues.

**FIGURE 1:**
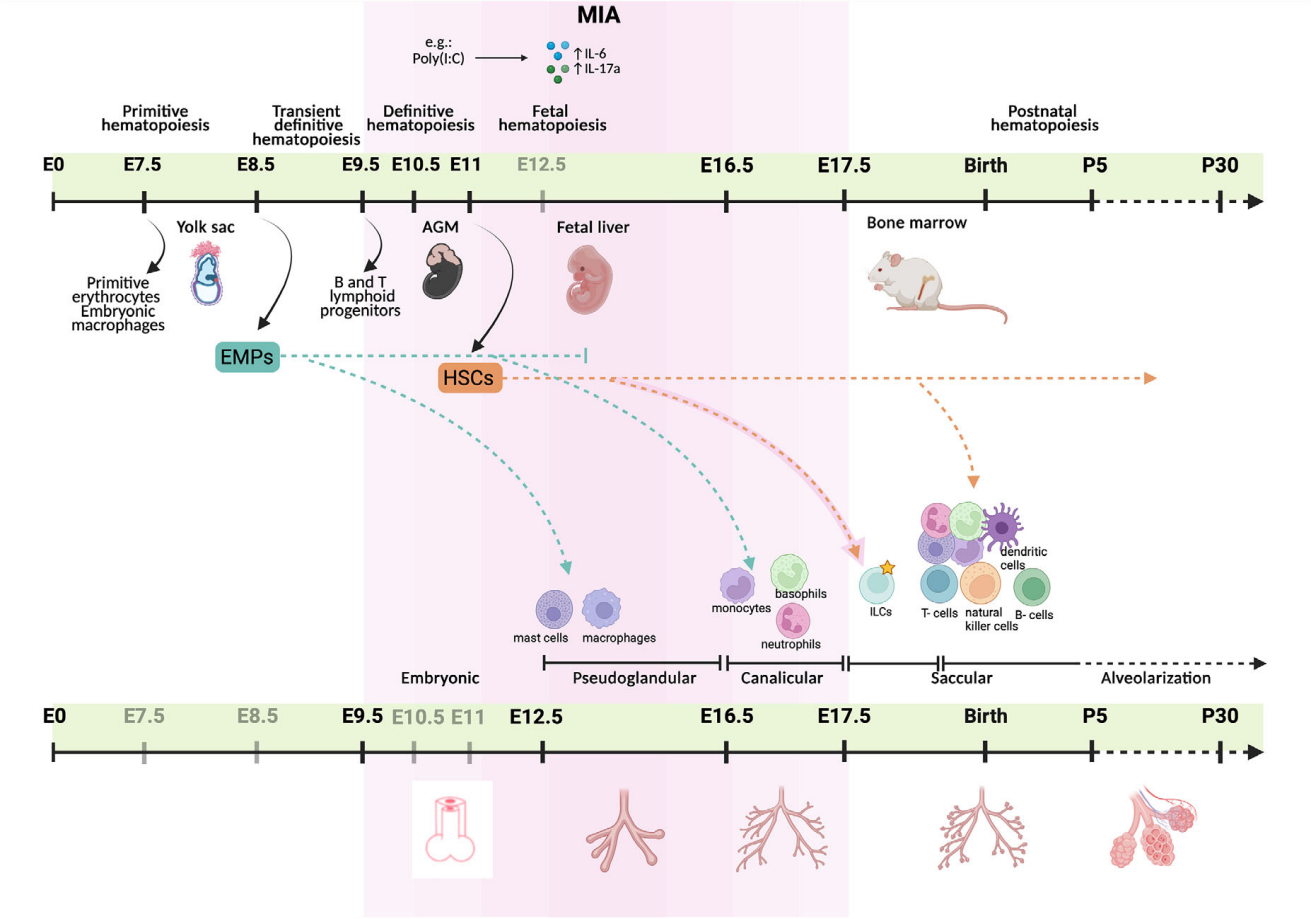
Overview of mouse hematopoietic stem cell and lung development. Hematopoiesis (upper timeline) starts with three main waves: primitive, transient definitive, and definitive hematopoiesis. During the first primitive wave, at E7.5, the first blood cells, such as primitive erythrocytes and embryonic macrophages, are produced to support the embryo. The second wave, the transient definitive wave, begins around E8.5. During this wave, EMPs (turquoise dashed line) emerge from hemogenic endothelial cells in the blood islands of the yolk sac. Immediately afterward, B and T's lymphoid progenitors also emerge from these cells in the yolk sac and the developing AGM region. Then, during the third definitive hematopoiesis, at E10.5–E11, the first HSCs (orange dashed line) emerge in the AGM region. The definitive HSCs then migrate to the fetal liver, which becomes the primary site for hematopoiesis until late gestation (fetal hematopoiesis). During late gestation and until after birth, hematopoiesis transitions to the bone marrow, becoming the primary site for blood cell production throughout adulthood (postnatal hematopoiesis). The development of the lung (lower timeline) begins in the embryonic phase (E9.5–E12.5), where the lung tissue develops from the anterior intestinal endoderm. The pseudoglandular phase (E15.5–E16.5) is characterized by massive branching morphogenesis. In this phase, the primary lung buds divide into a branching network of tree‐like bronchi and bronchioles. In addition, macrophages and mast cells, originating from the EMPs, colonize (turquoise dashed arrow). Airways and future blood capillaries are formed in the canalicular phase (E16.5–E17.5). The progressive degeneration of epithelial function prepares the lungs for subsequent gas exchange. Monocytes, neutrophils, and basophils, originating from the fetal liver's EMPs (turquoise dotted arrow), also colonize the lungs during this time. The saccular phase (E17.5–P5) is a time of further growth of the terminal airways, in which the first sac‐like bodies develop, which are precursors of the later alveoli. In addition, ILCs originating from HSCs (orange dashed arrow) of the fetal liver colonize the lung during the early saccular phase (around E17.5). ILCs are so far the only subgroup of immune cells for which MIA has been shown to directly affect their development or function during this critical window of lung maturation (star). At the same time, the invasion of numerous types of immune cells begins, all of which now originate from the HSCs of the bone marrow. During this phase, macrophages, monocytes, mast cells, neutrophils, basophils, dendritic cells, natural killer cells, T cells, and B cells colonize the lung tissue. The alveolar phase (P5–P30) is the last phase of lung development. During this period, the number of alveoli increases through septation, which leads to a remarkable increase in the gas exchange surface of the lungs, thus making them fully functional. One MIA model can be induced by poly(I:C), resulting in high expression levels of cytokines such as IL‐6 and IL‐17A. Although most studies induce MIA between E12.5 and E14.5, it can also be triggered between E9.5 and E17.5 (pink bar). MIA, maternal immune activation.

During fetal development in mice, hematopoiesis occurs in several stages, beginning in the yolk sac and AGM region and moving to the fetal liver and later the bone marrow. Hematopoiesis begins during early embryonic development, with primitive hematopoiesis as a first wave occurring in the yolk sac around embryonic day (E7.5–E8). At this stage, the first blood cells, such as primitive erythrocytes and embryonic macrophages, are produced to support the embryo. These cells are short‐lived but essential for immune defense, providing critical support for the embryo's survival before more mature hematopoietic processes take over. The second wave, the transient definitive wave, begins around E8.5. During this wave, erythromyeloid progenitors (EMPs) emerge from hemogenic endothelial cells in the blood islands of the yolk sac. B and T's lymphoid progenitors also emerge from these cells in the yolk sac and the developing AGM region. As development progresses, the process shifts to definitive hematopoiesis, beginning around E10.5–E11 in the AGM region. Here, the first HSCs emerge, capable of self‐renewal and giving rise to all blood cell lineages [53]. Studies have confirmed that HSCs originate autonomously in the AGM [54]. Unlike the transient primitive cells, these definitive HSCs are the foundation for the adult hematopoietic system, as they can maintain lifelong blood cell production. The definitive HSCs then migrate to the fetal liver, where fetal hematopoiesis occurs from E11 until late gestation. The fetal liver is the primary site for HSC expansion and differentiation in utero. In this supportive microenvironment, HSCs proliferate and mature, producing a diverse array of blood cells needed for the developing fetus. The fetal liver is essential for ensuring that the embryo has a functional immune system and sufficient blood cells at birth (Figure 1) [55].

### 5.2 Bone Marrow Hematopoiesis

Towards the end of gestation, hematopoiesis transitions to the bone marrow, becoming the primary site for blood cell production throughout adulthood. This phase, known as postnatal hematopoiesis, ensures continuous replenishment of blood and immune cells (Figure 1). Bone marrow serves as a home for HSCs, which are maintained in a niche and regulated by growth factors and cytokines such as granulocyte‐macrophage colony‐stimulating factor (GM‐CSF), IL‐6, and IL‐3 to control self‐renewal and differentiation [56].

In response to stress or injury, the body activates stress hematopoiesis. This adaptive response is caused mainly by stress hormones such as glucocorticoids and catecholamines, resulting in the rapid expansion of HSCs. Stress hematopoiesis allows the body to respond quickly to acute situations, like infection or blood loss, ensuring an adequate supply of immune and blood cells to restore homeostasis [57].

### 5.3 MIA‐Induced Long‐Term Immune Programming

Maternal inflammation, induced by poly(I:C) at E14.5 during pregnancy, has been shown to affect the development of fetal HSCs, leading to long‐term changes in the bone marrow and peripheral blood immune cell populations postnatally with sustained increase in the expansion of lymphoid‐biased fetal progenitors and an elevated output of lymphoid‐biased progenitors such as Fms‐like tyrosine kinase 2+ multipotent progenitor cells. Postnatally, this resulted in higher numbers of lymphoid‐derived immune cells, which could shape the immune system's trajectory and potentially influence susceptibility to immune‐related diseases later in life [58]. Additionally, inflammatory cytokines like IL‐6, typically produced during MIA, could play a significant role in this process, influencing HSC differentiation. It has been found that IL‐6 signaling skews HSC differentiation towards myeloid‐biased hematopoiesis. In a systemic lupus erythematosus model, IL‐6 inhibited B lymphopoiesis while promoting myeloid differentiation. Elevated IL‐6 levels in systemic lupus erythematosus‐prone mice were associated with reduced B cells and more primitive B‐lymphoid progenitors. Interestingly, uncommitted progenitors in these mice expressed the IL‐6 receptor and, in response to IL‐6, upregulated Inhibitor of DNA binding 1, a transcription factor that inhibits lymphopoiesis and promotes myelopoiesis [59]. Recent studies have also shown the important role of the placenta in this context. The placenta is not only a passive site for fetal hematopoiesis that provides a physical space where hematopoiesis happens but also an active regulator of HSC development, playing a more complex and dynamic role. The placenta harbors a large pool of fetal HSCs during midgestation, starting at E10.5 to E11, which co‐occurs with hematopoiesis in the AGM region and expands between E12.5 and E13.5 [60, 61]. Further studies are needed to explore the effects of MIA on HSC development, as this could provide deeper insights into the long‐term impacts of prenatal inflammation on immune system programming.

### 5.4 The Fetal Lung Development as a Site of Immune Cell Programming

The fetal lung plays a critical role in transitioning from primitive to definitive hematopoiesis by providing a supportive niche for HSCs through various signaling mechanisms. Although the specific signals from the lung microenvironment have not yet been identified, megakaryocytes are known to be present in the lung, where they are thought to influence hematopoiesis via signaling pathways involving thrombopoietin and transforming growth factor‐beta [62]. Studies suggest that fetal and adult lungs may harbor a reservoir of hematopoietic progenitor cells [63, 64]. Factors such as C–X–C motif chemokine ligand 12, stem cell factor, and angiopoietin‐1 are thought to contribute to maintaining hematopoietic niches in the lung—possibly across the lifespan. This underscores the dual importance of the lung as a central developmental environment for the hematopoietic and immune systems before birth and as a potential hematopoietic niche in adulthood. The fetal lung is also a critical organ for immune cell development, as it is one of the first sites where immune cells populate and begin to differentiate. The development of the fetal lung can be divided into five stages: embryonic (E9.5–E12.5), pseudoglandular (E12.5–E16.5), canalicular (E16.5–E17.5), saccular (E17.5‐postnatal day (P) 0), and alveolar (P0–P14/21) [65, 66].

The lung development begins on E9 in the mouse. It starts in the anterior endoderm of the foregut. It occurs by expressing the transcription factor NK2 homeobox 1 in endodermal cells on the ventral side of the anterior foregut endoderm. However, no clear evidence exists that immune cells exist in the lung at this phase. On E9.5, the trachea separates from the anterior foregut endoderm. Furthermore, fibroblast growth factors [67], as well as sonic hedgehog [68] and Wnt signaling pathways [69], regulate the budding of the trachea from the anterior foregut endoderm. By E12.5, the trachea completely separates from the esophagus (Figure 1).

The pseudoglandular phase (12.5–16.5), which incidentally coincides with the timepoint MIA is induced in most model systems, is characterized by branching morphogenesis. This process regulates branch formation in both lung buds, leading to a tree‐like network of airways with thousands of terminal branches [65, 66]. Additionally, the development of the CD45^+^ immune cell compartment in the lung begins with early colonization by macrophages and mast cells, which originate directly from the yolk sac [70, 71]. Macrophages arise from a Tie^2^^+^ cellular lineage that produces Csf1r^+^ EMPs, a population separate from HSCs. These EMPs develop in the yolk sac around E8.5 [71] (Figure 1). Mast cells also originate from EMPs. The progenitors appear to generate mast cells either by seeding peripheral tissues directly or by migrating first to the fetal liver, where they mature before dispersal. However, the exact contribution of each pathway is uncertain, and both pathways likely coexist during development [72]. Regarding the development of nonimmune cells (CD45‐ cells), the lung consists primarily of undifferentiated fibroblasts and contains a low number of epithelial precursor cells. In addition, a small proportion of pericytes and endothelial cells can be found in the lungs at this stage [70]. This is followed by the canalicular phase (E16.5–E17.5), in which the terminal branches of the airways constrict and groups of epithelial cells aggregate to form bladder structures, which later develop into alveoli to prepare the lungs for breathing after birth [73]. In this phase, monocytes make up the majority of immune cells, whereas only neutrophils and basophilic granulocytes, which originate from the fetal liver, colonize the lung [70] (Figure 1). Monocytes originate from EMPs, which are found in the yolk sac and colonize the fetal liver, where they mature into fetal monocytes through a *c*‐Myb‐dependent pathway [74]. Basophils were traditionally viewed as immune cells generated in the bone marrow, circulating in the blood, and entering the tissue only during inflammation [75]. Single‐cell studies, however, show that they are already present in the fetal lung as early as embryonic Day 16.5—even before functional bone marrow hematopoiesis occurs. This indicates a prenatal derivation from the fetal liver, similar to mast cells and tissue‐dwelling macrophages. They develop a tissue‐specific phenotype within the lung in response to local cytokines such as IL‐33 and GM‐CSF [70]. Neutrophils are derived from EMPs within the yolk sac and colonize the lungs between E16.5 and E17.5 [70, 76]. At the same time, the proportion of progenitor epithelial cells increases significantly compared to the pseudoglandular phase [77].

### 5.5 Development and Differentiation of Lung Macrophages

During the saccular phase, Ly6C^hi^CD11b^hi^ fetal monocytes colonize the developing lung from the fetal liver [16.6–17.5]. After birth, in the late saccular phase, the fetal monocytes differentiate into mature alveolar macrophages under the influence of the growth factor GM‐CSF [78]. Alveolar macrophages, typically characterized by the expression of CD11c^+^, Siglec‐F^+^, and low CD11b, play a critical role in lung development, surfactant homeostasis, pathogen clearance, and the regulation of immune responses [78]. In parallel, macrophages, mast cells, neutrophils, basophils, dendritic cells, natural killer cells, T cells, and B cells begin to infiltrate the lung (Figure 1). All of these immune cells are derived from HSCs in the bone marrow. Although T cells originate from the bone marrow, they undergo maturation in the thymus [70]. Additionally, innate lymphoid cells (ILCs) also start to infiltrate the lung tissue. These cells contribute to regulating the immune response and maintaining tissue homeostasis. ILCs originate from common lymphoid progenitors in the fetal liver, where their differentiation is regulated by IL‐7 and Notch signaling. ILCs come from the fetal liver during the early saccular phase because they are part of an early wave of immune cells generated before the bone marrow entirely takes over hematopoiesis. After migrating to the lung, ILC2s receive support for their final differentiation and functional maturation from mesenchymal platelet‐derived growth factor receptor alpha‐positive glycoprotein 38‐positive cells [79]. Notably, smooth muscle fibroblasts, characterized by Enpp2 expression, emerge during the saccular stage of lung development. At the same time, epithelial progenitors differentiate into alveolar epithelial type 1 cells, marked by Akap5 and comprising approximately 5% of nonimmune (CD45^−^) lung cells, and alveolar epithelial type 2 cells, marked by Lamp3 and comprising about 7% of the nonimmune compartment. Moreover, from this phase onward, the components of the nonimmune cell types begin to stabilize [77], and the alveoli complete maturation in the alveolarization phase (P0–P14).

### 5.6 Impact of MIA on Lung‐Resident Immune Cells

Until now, only two studies have reported the effects of MIA on offspring lung ILCs. The first demonstrated that prenatal inflammation caused by MIA leads to lasting changes in the innate immune system. A single poly(I:C) injection during mid‐pregnancy disrupted the development of lymphoid precursor cells within the fetal liver environment. This led to an increased number and frequency of ILC‐bound progenitor cells soon after MIA. Postnatally, the ILC2s were increased in the lungs and greatly expanded at Day 14 postnatally and in adulthood. The ILC2s were hyperactivated and produced more IL‐5 and IL‐13, but overall cytokine levels in the lung were not changed, showing cell‐intrinsic changes in ILC2s. Hyperactivation of these cells led to immune landscape remodeling in the lung, with other immune cell types proliferating, with ILC2s being the first responders [80]. The second study confirmed the findings and showed that prenatal inflammation due to poly(I:C) exposure at E14.5 led to long‐term expansion and hyperactivation of ILC2 cells in offspring lungs. This also led to increased production of IL‐5 and IL‐13 by ILC2s, causing eosinophilic inflammation, disruption of the lung's immune landscape, and increased susceptibility to asthma in adulthood. Hyperactivation was preprogrammed at the fetal progenitor cell level, namely, through a lymphoid‐biased developmentally restricted HSC of the fetal liver. The effects were maternal TLR3‐mediated type I interferon response‐dependent [81]. In addition to the imprinting of ILC2 cells, other immunocompetent cells in the lung could also be potential targets of MIA. This justifies the need to consider the influence of early immune exposures on cell type‐specific basis at different time points during lung development.

### 5.7 Outlook and Outstanding Questions

Recent global outbreaks of viral diseases such as Zika and Severe Acute Respiratory Syndrome Coronavirus 2, as documented in human studies, have emphasized the importance of not neglecting the concept of the “window of opportunity” and being aware of the potential enhanced vulnerability of fetal and early life [82, 83, 84]. This per se is an extremely broad topic not to be covered in one review as the “exposome” consists of numerous biosocial elements within our changing world, resulting in continuously ongoing adaptations. This should not be regarded as negative but rather as an expression of evolutionary hard‐wired capacity to adapt. Nevertheless, it is important for scientists from different disciplines to zoom in on this bird's eye perspective and interrogate the mechanisms that underlie these—sometimes maladaptations—to design preventive strategies and eventually targeted interventions. Areas for further research that we have identified for immunologists are summarized in the box.

**Major outstanding questions in maternal immune activation research**

Which precise gestational windows represent the highest vulnerability to maternal immune challenges in lung and bone marrow niches?

How does placental immune function modulate fetal hematopoietic stem cell (HSC) development and offspring immune programming?

What are the long‐term effects of MIA on fetal lung‐resident immune and nonimmune cell populations, and how do these changes impact susceptibility to respiratory diseases or allergies?

How do placental immune changes mediate long‐term offspring health outcomes?

What are the mechanisms underlying epigenetic reprogramming of fetal immune cells during MIA?

How does maternal microbiota composition influence fetal immune development?

What preventive or therapeutic interventions can be designed targeting these pathways?

What are the key human biomarkers predictive of adverse neuroimmune outcomes following MIA?

## Author Contributions

All authors contributed equally to the conceptualization of the review. Literature search and manuscript writing were performed by all authors. All authors reviewed, edited, and approved of the final manuscript.

## Conflicts of Interest

The authors declare no conflicts of interest.

## Data Availability

Data sharing is not applicable to this article as no datasets were generated or analyzed during the current study.
